# A Case of Insulin Autoimmune Syndrome in an Indian Male Taking Alpha-Lipoic Acid

**DOI:** 10.7759/cureus.43743

**Published:** 2023-08-19

**Authors:** Tanisha Sehgal, Uday Ohri, Naveen Mittal, Parmarth Attri, FNU Dishant

**Affiliations:** 1 Medicine and Surgery, Dayanand Medical College and Hospital, Ludhiana, IND; 2 Medicine, Dayanand Medical College and Hospital, Ludhiana, IND; 3 Endocrinology and Diabetes, Dayanand Medical College and Hospital, Ludhiana, IND

**Keywords:** hypoglycemia, insulin autoimmune syndrome, α-lipoic acid, insulin autoantibodies, hyperinsulinemic hypoglycemia

## Abstract

Insulin autoimmune syndrome [IAS, Hirata disease (HD)] is a rare cause of recurrent spontaneous hypoglycemic episodes, characterized by high serum insulin levels and high titers of autoantibodies against endogenous insulin. We report a case of a previously healthy Indian male presenting with recurrent episodes of hypoglycemia with no prior exposure to exogenous insulin. Regular glucose monitoring was done. Laboratory tests showed insulin >1000 μIU/mL and C-peptide levels of 12.8 ng/ml. The patient had high titers of insulin autoantibodies (IAA) (>100 units/mL; normal range: <10 units/mL), which indicated a diagnosis of IAS. The patient was consuming alpha-lipoic acid; sulfhydryl-containing compounds have been linked to IAS. This case report highlights the importance of IAA titers in first-line investigations for hypoglycemia in a non-diabetic patient with strikingly high blood insulin levels and discusses the potential relationship between IAS and alpha-lipoic acid.

## Introduction

Insulin autoimmune syndrome (IAS) or Hirata disease (HD) was first described by Hirata in 1970 [1]. It is characterized by spontaneous hypoglycemic episodes and a high titer of insulin autoantibodies (IAA) in patients who are not treated with insulin or oral hypoglycemic agents and have not had any pathological abnormality of the pancreas. IAS is difficult to distinguish from other causes of hypoglycemia; hence, titers of IAA remain the gold standard for diagnosis of the disease. It is a self-remitting disorder but may require aggressive immunosuppression [2]. Therefore, therapeutic management must be carefully planned and individually adapted for each patient.

## Case presentation

A 51-year-old Indian man was admitted to Dayanand Medical College & Hospital, Ludhiana due to repeated episodes of severe hypoglycemia for the past 20 days. These episodes occurred during the fasting and postprandial state. Hypoglycemic episodes were characterized by neurogenic and neuroglycopenic symptoms, including sweating, anxiety, palpitations, and lethargy. On detailed history, it was observed that the patient was taking multivitamins containing alpha-lipoic acid, which he had started one month prior to the onset of symptoms. There was no history of any autoimmune disease or recurrent viral infections. His family history was insignificant. The patient was non-diabetic and had not been exposed to insulin, insulin secretagogues, or any other medications associated with hypoglycemia. The physical examination of the patient was unremarkable.

Investigations

At the time of admission, his blood glucose level was 44 mg/dl, and critical samples for insulin, C-peptide, and cortisol were withdrawn. A dextrose 25% infusion was immediately started after withdrawing the sample. Hypoglycemic episodes recurred when the patient was admitted, and hence regular glucose monitoring was done. Laboratory investigations showed insulin levels of 1000 μIU/mL (normal range: 2.6-24.9 μIU/mL, C-peptide of 12.8 ng/mL (normal range: 0.8-3.8 ng/mL), and serum cortisol of 14.76 mcg/dL. Tests to measure thyroid function were within normal limits. Contrast-enhanced CT (CECT) abdomen revealed no pancreatic mass. The laboratory values were suggestive of IAS. Accordingly, IAA titers were measured using enzyme immunoassay; the finding of high IAA titers (>100 U/ml, normal: <10 U/ml) confirmed our diagnosis. The autoimmune panel for anti-nuclear antibodies, anti-neutrophil cytoplasm antibodies, anti-dsDNA antibodies, rheumatoid factor, and anti-thyroid peroxidase was negative. Other laboratory tests were within normal limits.

Treatment

During hospitalization at our center, the patient was managed with intravenous dextrose infusion and prednisolone 60 mg/day. He was advised to take small frequent meals, avoid simple sugars and increase complex carbohydrates to reduce postprandial hypoglycemia, and alpha-lipoic acid was discontinued. Blood glucose and electrolyte levels were continuously monitored. The dextrose infusion was slowly tapered off, following which the patient did not have any episodes of hypoglycemia. The patient was discharged. The dose of prednisolone was tapered off to 40 mg/day on follow-up after four weeks. Gradually, the dose of prednisolone was further tapered off to 20 mg/day and then discontinued, over a period of two months. The patient remained asymptomatic without any additional episodes of hypoglycemia.

## Discussion

Similar to the majority of cases, our patient exhibited symptoms consistent with IAS or HD for several weeks following the administration of a medication containing a sulfhydryl group. A subsequent assessment revealed persistent elevated levels of IAA, confirming the diagnosis of IAS.

IAS is a hyperinsulinemia hypoglycaemic disorder characterized by raised IAA titers in the absence of any pathological abnormality of the pancreas and prior exogenous insulin exposure [1]. This syndrome presents commonly in adults. Based on the available information, this condition is predominantly prevalent in Japanese populations, and up until the year 2020, there had been merely 28 documented cases reported in India [2]. The exact prevalence is disputed as it has probably been underestimated due to the lack of awareness of the disease, and difficulties in diagnostic workup, particularly in rural areas.

The presence of human leukocyte antigen (HLA)-DR4 and HLA-DRB1*0406 in East Asian patients and HLA-DRB1*0403 in Caucasian patients has been observed in IAS [2]. The mechanism of hypoglycemia in IAS is assumed to be due to high levels of IAA and is commonly associated with other autoimmune diseases such as Graves' disease, systemic lupus erythematosus, rheumatoid arthritis, and ankylosing spondylitis [3]. While its exact pathogenesis is unknown, the most accepted hypothesis is that the IAA binds to insulin and forms insulin-antibody complexes, rendering insulin ineffective and leading to postprandial hyperglycemia [2,3]. This stimulates insulin secretion. The subsequent release of biologically active insulin leads to a mismatch between blood glucose and free insulin concentration. Due to the increased half-life of autoantibody-bound insulin, the effects may be prolonged. Drugs of the sulfhydryl group have been found to interact with the disulfide bonds of insulin and increase its immunogenicity [1,4,5]. Viral infections can act as superantigens or molecular mimickers, triggering the production of autoantibodies [1].

The Endocrine Society has highlighted the importance of testing for IAA titers in non-diabetic adults with hyperinsulinemia hypoglycemia by including it among the first-line tests performed in such patients. In the majority of cases, IAA-mediated disorders exhibit a self-limiting course, wherein spontaneous resolution typically occurs within three to six months [4] from the initial diagnosis, subsequent to the cessation of the causative medication. Others may benefit from small, frequent, low-carbohydrate meals to avoid postprandial hyperglycemia and subsequent insulin spikes followed by hypoglycemia. Similar to other autoimmune-based disorders, short courses of corticosteroids (oral prednisolone) may be used as adjunctive immunosuppressants, while in severe cases, plasmapheresis (to reduce insulin autoantibody titers) and immunosuppressants, such as azathioprine and mycophenolate mofetil, have been tried in IAS [3]. Rituximab, an anti-CD20 monoclonal antibody, has emerged as a new treatment modality in life-threatening cases where there is a failure of response to steroids [3,6].

## Conclusions

IAS continues to be regarded as an uncommon condition that is primarily observed in Asian patients; however, there has been a discernible rise in its incidence. Contributing factors may include the widespread use of over-the-counter medications relative to the past decades. These are potential triggering factors in the pathogenesis of the disease. Hence, it is imperative to consider IAS in the differential diagnosis of recurrent episodes of hypoglycemia. The index of suspicion should be higher when encountering a patient with an autoimmune disease or who has recently used a medication containing a sulfhydryl group. The diagnostic approach to IAS is complex, and the measurement of IAA still remains the gold standard for differential diagnosis. Discontinuation of the offending drug, dietary modifications, and glucocorticoids remain the treatment of choice.
